# Disease progression of HIV-1 infection in symptomatic and asymptomatic seroconverters in Osaka, Japan: a retrospective observational study

**DOI:** 10.1186/s12981-015-0059-6

**Published:** 2015-05-22

**Authors:** Dai Watanabe, Sachiko Suzuki, Misa Ashida, Yuka Shimoji, Kazuyuki Hirota, Yoshihiko Ogawa, Keishiro Yajima, Daisuke Kasai, Yasuharu Nishida, Tomoko Uehira, Takuma Shirasaka

**Affiliations:** AIDS Medical Center, National Hospital Organization Osaka National Hospital, 2-1-14 Hoenzaka, Chuo-ku, Osaka City, Osaka 540-0006 Japan; Department of Nursing, National Hospital Organization Osaka National Hospital, Osaka, Japan

**Keywords:** HIV-1 infection, Seroconverters, Disease progression

## Abstract

**Background:**

Estimates of the interval from HIV-1 infection to disease progression may be affected by selection bias, and data concerning asymptomatic early seroconverters are limited. We examined the interval until disease progression in HIV-1 seroconverters in whom the timing of infection could be estimated within 1 year before diagnosis.

**Methods:**

Subjects included newly diagnosed patients at Osaka National Hospital between 2003 and 2010 who had either (1) symptomatic acute HIV-1 infection with a negative or intermediate reaction on Western blotting and a positive reaction on an HIV RNA test (symptomatic acute group) or (2) a positive reaction on Western blotting at diagnosis and a <1-year interval from the last negative HIV test until the first positive test. The latter was divided into symptomatic recent or asymptomatic recent groups based on the presence or absence, respectively, of any transient fever between the last negative and first positive tests. Disease progression was defined as a fall in the CD4 count to <350 cells/microL on 2 consecutive tests, the start of anti-HIV therapy, or the onset of AIDS-indicator diseases. Information was retrospectively collected from medical records.

**Results:**

Subjects included 210 patients: 91 in the symptomatic acute group, 72 in the symptomatic recent group, and 47 in the asymptomatic recent group. In the symptomatic acute (0.8 years) and symptomatic recent (2.2 years) groups, the Kaplan-Meier estimate of median interval until disease progression was significantly shorter than that in the asymptomatic recent group (2.9 years). Multivariate analysis by Cox’s proportional hazards test showed that the symptomatic acute group (vs. asymptomatic recent group: hazard ratio: 1.93; 95% confidence interval: 1.14–3.36; p = 0.0140) and a baseline CD4 count of <400 cells/microL (hazard ratio: 3.88; 95% confidence interval: 2.57–5.96; p < 0.0001) were independent prognostic factors associated with early disease progression.

**Conclusions:**

Symptomatic seroconversion was associated with early disease progression. Furthermore, the estimated median interval until the CD4 count was <350 cells/microL was only 2.9 years even in patients with asymptomatic seroconversion. These results suggest the importance of early diagnosis in early seroconverters.

## Background

Studies in the late 1980s reported that the asymptomatic phase persisted for about 10 years in patients infected with human immunodeficiency virus (HIV)-1 [1-4]. However, various observational studies and meta-analyses have indicated that the baseline CD4-positive T-lymphocyte count (CD4 cell count) of the patients infected with HIV-1 in recent years is lower than that in previous studies, and that the plasma HIV-1-RNA level at the set point is higher [5-10], suggesting the possibility that HIV-1 has become more virulent and the asymptomatic phase has been shorter [11,12].

After primary infection with HIV-1, symptoms such as fever, lymph node swelling, and headache appear in 40%–90% of patients [13]. Fever is the most common symptom related to primary HIV-1 infection [14-16]. A cohort study involving patients with symptomatic acute HIV-1 infection is one way to observe and evaluate the spontaneous course of HIV-1 infection. However, there are some limitations regarding observational studies involving patients with symptomatic acute HIV-1 infection. In these patients, the disease progression is more rapid than in those with asymptomatic acute HIV-1 infection [17-20]. In addition, the severity of acute HIV-1 infection is also associated with disease progression [15,16,18]. Therefore, the spontaneous history in patients with symptomatic acute HIV-1 infection does not always reflect that of HIV-1 infection overall. In a cohort study involving early HIV-1 seroconverters, the proportion of patients with symptoms was also high, suggesting the presence of a selection bias [12,18].

With the appearance of new anti-HIV drugs and an accumulation of evidence, it has been recommended that antiretroviral therapy (ART) should be introduced in patients with a high CD4-positive T lymphocyte count (CD4 cell count). In Japanese guidelines, the CD4 cell count cutoff for the start of ART also increased from 200 to 350 cells/μL in 2008 and then to 500 cells/μL in 2013. However, the estimated interval from HIV infection until the CD4 cell count decreases to 350 cells/μL is still important for evaluating the timing of ART initiation. Concerning recent reports [5,16,20-27] of the estimated interval until disease progression, limitations of selection bias may be present as described above. In addition, data concerning asymptomatic early seroconverters are limited. We therefore focused on the characteristics of HIV-1 seroconverters both with and without symptoms related to primary HIV-1 infection. In this study, patients newly diagnosed based on positive reactions on Western blotting were investigated. Those in whom the timing of HIV-1 infection could be estimated to have been within 1 year before the HIV-1 infection diagnosis were divided into 2 groups with respect to the presence or absence of a history of fever between the last negative and first positive tests. We examined the characteristics by comparing the clinical course between these 2 groups and patients diagnosed with symptomatic acute HIV-1 infection.

## Results

### Patient groups

Of 1199 patients newly diagnosed with HIV-1 infection between 2003 and 2010 at the National Hospital Organization Osaka National Hospital, in 210, the timing of HIV-1 infection could be estimated within 1 year before their diagnosis. Ninety-five symptomatic patients had a negative or intermediate reaction detected on Western blotting at the time of HIV-1 infection diagnosis, and the polymerase chain reaction (PCR) method showed a positive reaction. Of these, 4 were diagnosed with chronic infection based on the changes in the Western blotting results, clinical course, and absence of a recent risk of infection. Therefore, 91 were diagnosed with symptomatic acute HIV infection (symptomatic acute group). In 119 patients, a positive reaction was detected on Western blotting at the time of HIV-1 infection diagnosis and the interval from the last negative test until the first positive test was ≤1 year (recent group). Of these, 72 reported having a transient fever between the last negative and first positive tests (symptomatic recent group). The other 47 patients were assigned to the asymptomatic recent group.

### Patient characteristics and comparisons among symptomatic acute, symptomatic recent, and asymptomatic recent groups

Patient characteristics are shown in Table 1. The demographic and clinical characteristics of the subjects were compared among the symptomatic acute, symptomatic recent, and asymptomatic recent groups. There were no differences in the age or estimated route of infection among these 3 groups. In the symptomatic acute and symptomatic recent groups, the proportion of patients diagnosed in the late phase (2007–2010) was higher than that of patients diagnosed in the early phase (2003–2006). In the symptomatic recent group, 46 patients (64%) consulted a hospital for fever between the last negative and first positive tests. However, no HIV test was performed in any patient. The interval from the last negative test until the first positive test was compared between the symptomatic recent and asymptomatic recent groups. In the former (median 7.1 months), it was significantly shorter than in the latter (median 10.1 months), suggesting that a diagnosis of HIV infection was made earlier in the symptomatic recent group. Information regarding HIV-1 subtype was available in 111 of 210 participants. The most common subtype was B (n = 108), followed by CRF_01AE (n = 3). The percentage of patients with subtype B HIV-1 infection was not statistically different among the symptomatic acute, symptomatic recent, and asymptomatic recent groups (Table 1) and was not changed from the early phase (2003–2006, 55%) to the late phase (2007–2010, 49%) (p = 0.1900).

**Table 1:** **Demographic and clinical characteristics of participants**

**Characteristic**	**Symptomatic acute group**	**Symptomatic recent group**	**Asymptomatic recent group**	**p value**
Number of participants	91	72	47	
Age at first visit (y), median [IQR]	33 [28–38]	30 [25–35]	31 [24–36]	0.0841
Men, n (%)	91 (100%)	69 (97%)	47 (100%)	0.0387
Estimated route of transmission, n (%)				
Homosexual	79 (87%)	66 (92%)	43 (92%)	0.3234
Heterosexual	9 (10%)	6 (8%)	2 (4%)	
Unknown	3 (3%)	0 (0%)	2 (4%)	
HIV-1 subtype				
B	41 (45%)	40 (56%)	27 (57%)	0.0983
CRF01_AE	0 (0%)	1 (1%)	2 (4%)	
Not determined	50 (55%)	31 (43%)	18 (38%)	
Nationality, n (%)				
Japanese	89 (98%)	70 (97%)	47 (100%)	0.3490
Other	2 (2%)	2 (2%)	0 (0%)	
Calendar year at diagnosis, (%)				
2007–2010	65 (71%)	45 (63%)	20 (43%)	0.0044
2003–2006	26 (29%)	27 (37%)	27 (57%)	
Medical exam between last negative and first positive test, n (%)				
No	3 (3%)	26 (36%)	47 (100%)	<0.0001
Outpatient	28 (31%)	36 (50%)	0 (0%)	
Hospitalization	60 (66%)	10 (14%)	0 (0%)	
Interval between last negative and first positive test (mo), median [IQR]		7.1 [4.1–10.2]	10.1 [6.1–11.7]	0.0472
Baseline CD4 cell count (cells/μL), median [IQR]	359 [243–464]	353 [257–481]	445 [353–526]	0.0323
Percentage of patients with CD4 cell count <400 cells/μL, n (%)	54 (59%)	43 (60%)	15 (31%)	0.0035
Baseline HIV-1 RNA level (copies/mL), median [IQR]	470000 [52000–270000]	32000 [6900–74800]	21000 [12000–66000]	<0.0001
Percentage of patients with HIV-1 RNA level ≥50000 copies/mL, n (%)	69 (76%)	26 (36%)	14 (30%)	<0.0001

Abbreviation: IQR, interquartile range

### Comparison of baseline CD4 cell count and plasma HIV-1 RNA level

We examined the CD4 cell count and viral level on the initial consultation. Overall, the median CD4 cell count and plasma HIV-1 RNA level were 383 cells/μL and 51,900 copies/mL, respectively. The proportion of patients with a CD4 cell count of <400 cells/μL and that of patients with a plasma HIV-1-RNA level of ≥50,000 copies/mL are presented in Table 1. In the symptomatic acute and symptomatic recent groups, the proportions of patients with a baseline CD4 cell count of <400 cells/μL were higher than that in the asymptomatic recent group (symptomatic acute vs. asymptomatic recent: p < 0.01; symptomatic recent vs. asymptomatic recent: p < 0.01). Furthermore, the proportion of patients with a baseline plasma HIV-1-RNA level of ≥50,000 copies/mL was the highest in the symptomatic acute group among the 3 groups (symptomatic acute vs. asymptomatic recent: p < 0.001; symptomatic acute vs. symptomatic recent: p < 0.001).

### Estimated interval from HIV-1 infection until disease progression

**Figure 1:**
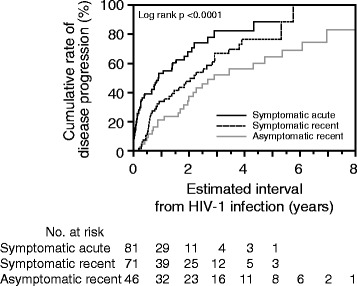
Kaplan-Meier plot of the cumulative rate of disease progression in the study participants. Comparison of the cumulative rate of disease progression among the symptomatic acute, symptomatic recent, and asymptomatic recent groups.

**Figure 2:**
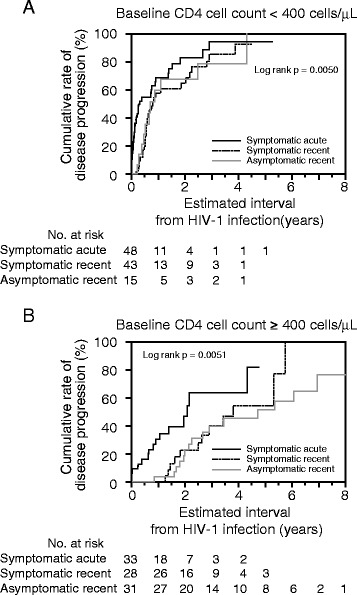
Kaplan-Meier plot of the cumulative rate of disease progression stratified with the CD4 cell count. **(A)** Patients with a CD4 cell count of <400 cells/μL on the initial consultation. **(B)** Patients with a baseline CD4 cell count of 400 cells/μL or more.

We examined the interval from the estimated timing of infection until disease progression. Twelve patients enrolled in a clinical study, in whom ART was started regardless of their CD4 cell counts, were excluded from the analysis. Of the 198 patients, 119 met the criteria for disease progression during the follow-up period (median 0.78 years). Of these, 96 patients showed a CD4 cell count of <350 cells/μL on 2 consecutive tests. In the other 19 patients who had a CD4 cell count of <350 cells/μL on at least 1 test, ART was started (9 in the acute symptomatic phase and 6 in the chronic asymptomatic phase) or AIDS-indicator diseases were diagnosed before the above criterion was met. In the remaining 4, although the CD4 cell count did not reach <350 cells/μL on either test, ART was started for prevention of partner infection, HBV infection, thrombocytopenia, and HIV infection-related malaise in each patient. In the symptomatic acute group, in only one of the patients with a CD4 cell count of <350 cells/μL on the first and second tests did the CD4 cell count return to ≥350 cells/μL within the next 3 months. Using the Kaplan-Meier method, we estimated the interval from the estimated timing of infection until disease progression. In the cohort overall, the median interval until disease progression was 1.9 years. In the symptomatic acute, symptomatic recent, and asymptomatic recent groups, it was 0.8, 2.2, and 2.9 years, respectively (Figure 1), showing significant differences (symptomatic acute vs. symptomatic recent: p < 0.001; symptomatic acute vs. asymptomatic recent: p < 0.001; symptomatic recent vs. asymptomatic recent: p < 0.05). Using Cox’s proportional hazards model, we investigated factors involved in early disease progression (Table 2). On univariate analysis, a baseline CD4 cell count of <400 cells/μL, and belonging to the symptomatic acute group were significant factors. Multivariate analysis showed that the baseline CD4 cell count and belonging to the symptomatic acute group were independent factors. To verify the results of multivariate analysis, we conducted subgroup analysis by stratification of the CD4 cell count on the initial consultation (Figure 2). In the symptomatic acute group, disease progression was observed in the early stage regardless of the baseline CD4 cell count. However, the interval until disease progression was similar between the symptomatic recent and asymptomatic recent groups through stratification. These results showed that the interval from HIV infection until disease progression was associated with the CD4 cell count on the initial consultation, a diagnosis of acute HIV infection, and fever suggestive of primary infection with HIV-1 and that the former 2 were independent factors.

**Table 2:** **Association with early disease progression in early HIV-1 seroconverters**

	**Univariate results**			**Multivariate results**		
	**HR**	**95% CI**	**p value**	**HR**	**95% CI**	**p value**
Age (≥40 vs. <40)	1.21	0.73–1.93	0.4411	1.25	0.75–2.00	0.3739
Calendar year at diagnosis (2007–2010 vs. 2003–2006)	1.36	0.93–2.03	0.1170	1.26	0.83–1.93	0.2823
With baseline CD4 cell count <400 cells/μL	4.02	2.70–6.08	<0.0001	3.88	2.57–5.96	<0.0001
Group						
Asymptomatic recent group	1			1		
Symptomatic recent group	1.62	0.97–2.74	0.0621	1.12	0.67–1.93	0.6772
Symptomatic acute group	2.95	1.79–4.99	<0.0001	1.93	1.14–3.36	0.0140

Abbreviations: HR, hazard ratio; CI, confidence interval.

## Discussion

This study was conducted in HIV-1 seroconverters in Osaka (Japan) in whom the timing of infection could be estimated within 1 year before diagnosis. Initially, as important observation points, disease progression was noted in the early stage in symptomatic HIV-1 seroconverters. In those diagnosed in the acute stage of HIV-1, the median interval until disease progression was only 0.8 years (Figure 1). Although some studies have estimated the interval from infection until the CD4 cell count reaches <350 cells/μL in patients with acute HIV-1 infection [5,16,20-24,26,27], it is difficult to compare the results among different cohorts due to differences in the definition of acute HIV-1 infection and disease progression or patient background. However, similar median intervals have been reported from Japan (<1 year) [16], Holland (<1 year) [24], and Germany (8.3 months) [26], although some reports showed longer median intervals than this report does [5,20,21,23,27]. The reasons suggested for early disease progression include host-related factors such as genetic background, route of infection, and social background; viral factors such as subtype and escape mutations; and the possibility of a selection bias in which severe-status patients were predominantly enrolled in the study [28,29]. The mechanism of early disease progression remains to be clarified, but it must be considered that disease progression is rapid in patients with symptomatic acute HIV-1 infection.

Even in patients without primary infection symptoms (asymptomatic recent group), the median interval from infection until disease progression was 2.9 years. In a large-scale observational study involving 18,495 seroconverters, the interval from infection until the CD4 cell count reached <350 cells/μL was estimated to be 4.19 years [25]. However, approximately 50% of the study subjects had been diagnosed with HIV-1 infection before 1996. The subjects included patients with symptomatic acute HIV infection. Therefore, although the interval from infection until the CD4 cell count reached <350 cells/μL is longer than what we report here, a direct comparison between the previous report [25] and ours is not possible.

Of the 119 patients with positive reactions on Western blotting at the time of HIV-1 infection diagnosis, symptoms possibly related to primary infection with HIV-1 were observed in 72. However, this figure may include an error. Although patients were questioned about a history of fever at the initial consultation, fever is not always related to primary HIV-1 infection. Furthermore, we cannot rule out the possibility that some patients could not recollect the appearance of symptoms related to primary infection and were assigned to the asymptomatic recent group. However, when analyzing the HIV-1 RNA level (Table 1) and interval from infection until disease progression (Figure 1), the symptomatic acute and asymptomatic recent groups showed opposite characteristics. Those of the symptomatic recent group were intermediate between the other 2 groups. Based on these results, we believe the majority of participants were assigned to the correct groups. Several possibilities may explain why the symptomatic acute and symptomatic recent groups showed different disease progression. One possible reason is the difference in disease severity because the proportion of patients who were hospitalized in the symptomatic acute group was higher than that in the symptomatic recent group (Table 1). However, we cannot exclude the possibility that recall bias, as mentioned above, may have affected this difference.

As shown in Table 1, the interval from the last negative test until the first positive test in the symptomatic recent group was significantly shorter than in the symptomatic recent group. This suggests the possibilities that the development of fever motivated those who recognize the risk of HIV-1 infection to undergo voluntary HIV testing and that the true seroconversion date with symptomatic seroconverters would be closer to the date of the first positive test rather than the date of the last negative test. These possibilities were also indicated by the recent report estimating the date of infection for HIV-1 infected patients using the HIV-1-specific immunoglobulin G (IgG) levels measured by the IgG-capture BED-enzyme immunoassay [30].

A limitation of this study is that it was a retrospective analysis involving a limited number of patients in a single institution. We are also aware of possible limitations of selection and recall biases in this study. Selection of the patients with fever related to the primary HIV-1 infection could be a potential source of selection bias. However, the data presented in this study are consistent with the results of previous studies. Patients diagnosed with acute HIV-1 infection showed disease progression in the early stage. Fever related to primary HIV-1 infection encouraged patients undergo HIV testing. Additionally, disease progression may be observed within a relatively short period even in asymptomatic early-infection patients.

## Conclusions

Symptomatic HIV-1 seroconversion was associated with early disease progression, especially in patients with symptomatic acute HIV-1 infection, with a median interval until disease progression of only 0.8 years. Furthermore, the estimated median period during which the CD4 cell count was <350 cells/μL was only 2.9 years even in patients with asymptomatic seroconversion. These results suggest the importance of early diagnosis in early HIV-1 seroconverters.

## Methods

### Case selection

This study was conducted at the National Hospital Organization Osaka National Hospital, a major hospital for HIV infection in Japan, located in the center of Osaka. Subjects included newly diagnosed patients with HIV-1 infection who consulted this hospital for the first time between 2003 and 2010 and who met 1 of the following 2 criteria: (1) a negative or intermediate reaction detected on Western blotting at the time of HIV-1 infection diagnosis and a positive PCR, leading to a diagnosis of symptomatic acute HIV-1 infection (symptomatic acute group) or (2) a positive reaction detected on Western blotting at the time of HIV-1 infection diagnosis and a <1-year interval from the final negative HIV test until the first positive HIV test (recent groups). The protocol of this study was approved by the Ethics Review Board of the National Hospital Organization Osaka National Hospital (Approval No. 0913).

### Measurement of the CD4 cell count, plasma HIV-1 RNA level, and Western blotting

The CD4 cell count was measured by flow cytometry using the single-platform method. Peripheral blood (100 μL) was incubated with 10 μL of CYTO-STAT tetraCHROME reagent (Beckmann Coulter, Miami, FL) containing CD45-FITC/CD4-RD1/CD8-ECD/CD3-PC5 monoclonal antibodies. After erythrocyte hemolysis and cell fixation with the whole blood lysing reagent Kit (Beckmann Coulter), 100 μL of Flow-Count fluorospheres were added to the sample, and the absolute CD45 + CD3 + CD4 + CD8– cell counts were measured using a Cytomics FC500 system (Beckmann Coulter). The plasma HIV-1 RNA level was determined using the reverse-transcription PCR method (COBAS AMPLICOR HIV-1 test or COBAS TaqMan HIV-1 test; Roche Molecular Diagnostics, Branchburg, NJ). The CD4 cell count and plasma HIV-1 RNA level were measured at 1- to 4-month intervals. For Western blotting for HIV-1, LAV Blot I (Bio-Rad Laboratories, Hercules, CA) was used. Interpretation of the results was performed according to the criteria established by the World Health Organization.

### Definition of the symptomatic acute, symptomatic recent, and asymptomatic recent groups and comparison of clinical information on the initial consultation among the groups

The symptomatic acute group was defined as described above. Patients meeting conditions for both the symptomatic acute and recent groups were assigned to the symptomatic acute group. In the recent group, any self-reported history of a transient fever was investigated at the initial consultation in our hospital. Fever was defined as a body temperature of 38°C or higher. When a definitive diagnosis of a disease that causes fever was made, the patient was excluded from a history of fever. Patients who recollected having a transient fever between the last negative and first positive tests for HIV infection were assigned to the symptomatic recent group, and those who did not were assigned to the asymptomatic recent group. Information on the age, sex, nationality, estimated route of infection, year of diagnosis, previous hospital consultations, CD4 cell count, and plasma HIV-1 RNA level was collected from medical records and compared among the 3 groups.

### Estimation of the timing of HIV-1 infection and interval from infection until disease progression

In the symptomatic acute group, the timing of infection was estimated to be 1 month before a diagnosis of HIV-1 infection was made. In the recent groups, the midpoint between the last negative and first positive tests was regarded as the estimated timing of infection. The timing of disease progression was defined as the earliest date on which the CD4 cell count was less than 350 cells/μL on 2 consecutive tests conducted at more than 1 month apart, the start of ART, or the onset of an AIDS-indicator disease. In patients in whom the annual frequency of testing for the CD4 cell count and plasma HIV-1 RNA level became <3, follow-up was completed on the final day of testing. The interval from the estimated timing of infection until disease progression was calculated using the Kaplan-Meier method. For significance tests, the log-rank test was performed. When there was a significant difference, adjustment was conducted using Holm’s correction, and multiple tests were performed. For multivariate analysis, Cox’s proportional hazards analysis was carried out. With respect to individual factors, the hazard ratio, 95% confidence interval, and p-value were calculated.

### Statistical analysis

For 3 × 2 cross-table tests, the *χ*^2^ test was used. When there was a significant difference, adjustment was conducted using Holm’s method, and multiple tests were performed. To compare the results among the 3 groups, analysis was conducted using Kruskal-Wallis one-way analysis of variance by ranks. The Kaplan-Meier method, log-rank test, and Cox’s proportional hazards test were performed, as described above. For statistical analysis, JMP version 10.0.0 software (SAS Institute, Cary, NC) was used. A p-value of <0.05 was regarded as significant.
